# Monthly Summary

**Published:** 1892-10

**Authors:** 


### Monthly Summary.

INFLUENCE OF HEREDITY.—*The Medical Record* gives an account of a series of experiments undertaken by Dr. C. G. Lockwood, of New York, to determine the power of transmission of mutilations in animals. White mice were selected, because they begin to breed when but thirty days old. They were bred "in and in" for ninety-six generations, all the sickly and defective ones being killed. Pursuing this course, notwithstanding the close breeding, at the end of that time the animals were larger and finer than the first ones.

The tails of the original pair were cut off. When they had young, a strong pair of these were selected, their tails cut off, and they were put in a cage by themselves. A pair of their progeny were selected, and the same course pursued. Thus in each instance brother and sister were the ones chosen for parents of the next litter, and in every case their tails were clipped. In seven generations young mice began to appear without tails, and he finally produced a perfect breed of tailless mice.

He then commenced the reverse process, by crossing a mouse without and one with a tail, alternating the sexes of each generation, and finally he got a breed of full-tailed mice again. Thus a persistence in removing the tails, and continued in-breeding of the same families, produced mice without tails; but when these were crossed with mice of the same origin, but possessing tails, there was a return to the ancestral condition, the hereditary tendency toward mice with tails being much the stronger.

Many experiments have been tried to determine the force of transmission of parental peculiarities. All experience teaches that bodily and mental traits are inherited. All breeders of animals know that it is possible materially to vary a type by judicious crossing and persistent in-breeding. But the permanence of such variation is a matter of speculation. If all horses were allowed to run wild, there is little doubt that the race horse and the cart horse would soon lose their distinctive traits, and there would be a tendency toward the original type. So, if all dogs were withdrawn from man's dominion and care, there would be a universal degeneration toward their wolfish ancestral traits. There are few who do not admit that varieties and species may have been produced by peculiar environments, and that the long contiuued disuse or destruction of any organ will finally result in its disappearance. Yet there are some stubborn facts which refuse to conform to the supposed general rule, and among these none is more striking than the persistence of the hymen, notwithstanding its long continued obliteration.—*Dental Pract. and Advertiser.*

---

MAL-OCCLUSION OF ARTIFICIAL MOLAR TEETH.— Sometimes dentists find a great deal of difficulty in properly articulating artificial teeth. When the denture is completed, it is found that the molars strike too quickly, while the anterior teeth do not occlude at all. It becomes necessary to grind off the molars until the cusps are entirely removed, and a disagreeably smooth surface, unfit for mastication, is the result. Often, too, there is an annoying "click" when the teeth are brought together.

Usually this is the result, either of crowding the anterior portion of the wax articulating model beyond the point at which it rested when the bite was complete, or of allowing the posterior portion to rise, either of which will make the plaster casts of the occluding molars too short. The "clicking" is due to imperfect occlusion.

The undue length of the molar teeth may be, in some instances, owing to other causes, such as an improperly proportioned articulation, or to the springing of the posterior portions of the plate when a rubber base is used, but the most common cause is that first quoted.

Many a perfect fitting plate has been made almost useless by presenting two inclined planes upon occluding teeth. It is loosened by sliding backward or forward down this inclined surface, while perfect mastication is effectually prevented. More dentures fail by reason of bad occlusion, than through a poor fit.—*Dental Pract. and Advertiser.*

---

ETHER VERSUS CHLOROFORM.—The conclusions of the author, based upon fifteen years' experience with chloroform and fourteen years' experience with ether, may be briefly summed up as follows:

First, that ether is far less dangerous than chloroform.

Second, that the anæsthesia produced by ether is equally as complete and profound as that occasioned by chloroform.

Third, that the inconveniences of ether, absent in chloroform, may be greatly lessened by a proper administration of the drug.

Fourth, that the only contraindications for ether are its inflammability, and its action upon the respiratory mucous membranes in cases where these are diseased or are in bad condition.

The mask used by Julliard for the administration of ether resembles the simple chloroform mask with the following modifications: the outer portion is covered with some impermeable cloth, either mackintosh or rubber, to prevent the too rapid evaporation of the drug; it is also sufficiently large to permit of free respiration. The inhaler or mask is 15 centimetres long, 12 wide and 15 deep.

So much for Julliard's experiences and experiments. But he is not alone among Swiss and German surgeons in advocating the use of ether. Among German physicians in particular, who for a long time almost exclusively used chloroform as an anæsthetic, the gradual change of feeling is most marked. Garre, in the *Munch. Med. Wochenschrift*, gives the results of his experiments with both anæsthetics in a large number of cases, and heartily endorses the use of ether as a surgical anæsthetic in preference to chloroform. In 400 surgical cases in which ether was used as an anæsthetic, he observed that in those patients suffering from an organic lesion of the heart the drug was well borne and even exerted a beneficial action upon the affected organ; while in cases of respiratory troubles the irritative properties of ether rendered its use very objectionable. He concludes, therefore, that ether is preferable to chloroform in patients suffering from cardiac troubles; but on the contrary that chloroform is preferable to ether in cases of bronchial catarrh and similar affections. Garre found that anæsthesia occurs on an average in about four minutes, and that about 80cc. are required to maintain anæsthesia for a half an hour.

Dr. Frieter, in a recent issue of the *Deutsche Zeit. fur Chirurgie*, also pleads the cause of ether as a general surgical anæsthetic; and Dr. Butter, in the *Archiv. fur Klin. Chirurgie*, speaks most highly of ether as a substitute for chloroform in general surgery.

A great advantage of ether over chloroform, as recently pointed out by these investigators, is the rapidity with which patients recover from the effects of ether as compared with chloroform. Death from anæsthetization syncope, they also claim, will almost be unknown should ether be used instead of chloroform.

Thus an anæsthetic, the superior merits of which have long been recognized by the surgeons in this country, is now only gradually asserting its claims among our professional brethren abroad.—EDITORIAL, *Med. and Surg. Reporter*.

DISINFECTION OF THE HANDS.—Dr. H. A. Kelly, ("American Journal of Obstetrics," December, 1891), after giving the details of a large number of experiments made by himself and his associates, gives the following conclusions as to the best method of disinfecting the hands:

Scrubbing the hands with especial attention to the nails,—not more than one millimetre in length,—for ten minutes in water, frequently changed, at about 104° F. Immersion of the hands in a solution of permanganate of potassium, made by adding an excess of the salt to boiling distilled water, until every part of the hands and lower forearms is stained a deep mahogany red or almost black color. They are then transferred at once to a saturated solution of oxalic acid until completely decolorized and of a healthy pink color. Washing off the oxalic acid in warm sterilized water.

By this simple process the hands are rendered more nearly absolutely aseptic than by any other known means.

The author has found that it is impossible to get rid of staphylococci by scrubbing the hands and nails from ten to twenty-five minutes with a sterilized brush, soap and water, temperature 104° F. The bichloride of mercury solutions as used, up to 1 : 500, are not germicidal, as supposed. Previous erroneous conclusions as to the efficiency of the bichloride are shown to be due to an inhibiting action which may persist at least twenty-four hours after the last use of the drug. Hydrogen peroxide and lysol (four per cent.) were tested and found wanting.

In the present state of our bacteriological knowledge as to the causes of inflammation and suppuration, we are bound to use every means in our power to avoid sowing any unnecessary germs in our wounds. Soap and water are, Dr. Kelly believes, the best disinfectants, if we use but one, for they remove all germs which will come away easily. The bichloride of mercury, although dangerous on wounds on account of its property of coagulating and causing necrosis of albuminous tissues, has the valuable property of inhibiting these germs with which it comes into contact. Permaganate of potassium and oxalic acid are harmless to the hands and are germicidal. Soap and water plus the permaganate of potassium and oxalic acid are the only true germicides, and therefore the best disinfectants we possess to-day.—*Medical and Surgical Reporter.*

---

VERY RARE INDEED.—It is a rare thing for a human being to have three sets of teeth, and seldom that a baby is born with teeth; but a growth of teeth after death is, perhaps, the most odd of all such freaks. A case of this kind is reported from Cincinnati. Some time ago, the body of Mrs. Cathrine Davis was exhumed for removal. She died in 1852, aged thirty-nine years. She had been entirely destitute of teeth for several years before her death. The coffin, when opened revealed the features in a perfect state of preservation. But, beyond this, and much more to the astonishment of the assembled relatives, the half-opened mouth seemed almost smiling with a full and nearly perfect set of teeth of quite an inch in length.—*Exchange.*

What kind of soil is that which grows teeth an inch long in the mouths of dead people? No one has yet been able to produce them in the edentulous jaws of living persons, but here are all the minute particulars of a full set developed after death. What excuse has the editor of a grave professional journal, supposed to be published for educational purposes, for inserting such a roaring absurdity as that? Is he credulous enough to believe it to be a scientific fact, and if not, what reason can there be for the repeating of such a preposterous yarn?—*Dental Practitioner and Advertiser.*

---

DR. EUGENE S. TALBOT contends that "the constant extraction of the permanent teeth from one generation to another before the osseous system has been fully completed, is developing a race of individuals with small jaws." The time is rapidly approaching when extracting teeth, unless an absolute necessity, will be a statutory offence.

